# Integrative Omics Analysis of Rheumatoid Arthritis Identifies Non-Obvious Therapeutic Targets

**DOI:** 10.1371/journal.pone.0124254

**Published:** 2015-04-22

**Authors:** John W. Whitaker, David L. Boyle, Beatrix Bartok, Scott T. Ball, Steffen Gay, Wei Wang, Gary S. Firestein

**Affiliations:** 1 Department of Chemistry and Biochemistry, University of California San Diego, La Jolla, California, United States of America; 2 Department of Cellular and Molecular Medicine, University of California San Diego, La Jolla, California, United States of America; 3 Division of Rheumatology, Allergy and Immunology, University of California San Diego, La Jolla, California, United States of America; 4 Department of Orthopaedics, University of California San Diego School of Medicine, La Jolla, California, United States of America; 5 Department of Rheumatology, University Hospital Zürich, CH-8091, Zurich, Switzerland; CEA - Institut de Genomique, FRANCE

## Abstract

Identifying novel therapeutic targets for the treatment of disease is challenging. To this end, we developed a genome-wide approach of candidate gene prioritization. We independently collocated sets of genes that were implicated in rheumatoid arthritis (RA) pathogenicity through three genome-wide assays: (i) genome-wide association studies (GWAS), (ii) differentially expression in RA fibroblast-like synoviocytes (FLS), and (iii) differentially methylation in RA FLS. Integrated analysis of these complementary data sets identified a significant enrichment of multi-evidence genes (MEGs) within pathways relating to RA pathogenicity. One MEG is Engulfment and Cell Motility Protein-1 (*ELMO1*), a gene not previously considered as a therapeutic target in RA FLS. We demonstrated in RA FLS that *ELMO1* is: (i) expressed, (ii) promotes cell migration and invasion, and (iii) regulates Rac1 activity. Thus, we created links between *ELMO1* and RA pathogenicity, which in turn validates *ELMO1* as a potential RA therapeutic target. This study illustrated the power of MEG-based approaches for therapeutic target identification.

## Introduction

Rheumatoid arthritis (RA) is a chronic inflammatory disease that primarily affects diarthrodial joints [1]. The synovial membrane is infiltrated by inflammatory cells, and the synovial intimal lining becomes hyperplastic, due in part, to increased numbers of fibroblast-like synoviocytes (FLS) [2]. These cells produce matrix metalloproteinases and pro-inflammatory cytokines that participate in the pathogenesis of disease. Furthermore, they exhibit a unique aggressive phenotype that contributes to joint damage and perpetuation of disease. Numerous mechanisms have been implicated in the invasive behavior of RA FLS, including abnormal sumoylation, increased expression of genes that favor cell survival, and somatic mutations of key genes [3]. Most recently, a stable RA FLS DNA methylation signature was reported and analysis implicated many pathways involved in immune function, cell adhesion, and cell migration [4].

Genome-wide association studies (GWAS) identify sequence variants that are linked to disease by comparing the genomes of cases and controls. These studies may uncover genes that influence disease susceptibility and risk; however, many human diseases are highly multifactorial with individual variants having small individual influences. For example, ~4.6% of RA risk variance can be explained by sequence variation in the most influential gene, HLA-DRB1; however, the cumulative influence of 2,231 weaker variants accounts for ~18% of risk variance [5]. GWAS have shown that immune-mediated diseases, including RA, are associated with many overlapping variants but the relationships are complex with variants within the same region often differing [6]. A limitation of GWAS of complex diseases is that they provide no information about the cell-type in which the identified genes drive disease. With RA additional genome-wide assays are needed to assign disease drivers to the cell-type where they have their effect.

Transcriptomic studies measure the mRNA levels of all genes and can be used to identify genes that are differentially expressed between control and disease. When transcriptomics is used to study the differential expression of genes in RA FLS, several thousand genes are identified [7]. Recently, genome-wide approaches have been increasingly applied to the study of DNA methylation [8]. In particular, specific alterations in DNA methylation are necessary for correct during human development and can occur during the progression of cancer [9,10]. A specific pattern of DNA methylation has also been identified that can segregate RA FLS from osteoarthritis (OA) or normal FLS [11]. Furthermore, the RA FLS DNA methylation signature, which includes at least 2,375 genes, is stable for multiple passages and reflects pathogenic phenotype [4]. While all of these genes might have an influence over the FLS RA phenotype, it is difficult to identify the most influential subset in isolation.

Some limitations of individual genome-wide assay can potentially be overcome through the layering of results from multiple genome-wide assays [12]. The cell types where disease-associated variants might drive disease can be identified by comparing with histone modification profiles that mark that cell lineage-specific regulatory elements [8,13]. To better understand the relationships that exist between disease associated genes, they can be painted onto gene interaction networks, such as protein-protein interaction networks [14,15]. However, these strategies have not yet been applied to RA FLS.

Therefore, we performed an integrative analysis of epigenome, transcriptome and sequence variation in RA FLS to prioritize genes for therapeutic targets. We first established sets of genes implicated in RA using these three genomics approaches in isolation. Then we overlapped these sets to identity multi-evidence genes (MEGs). One MEG, namely *ELMO1* [16], was identified and validated in cultured FLS as potential participant in the pathogenesis of RA. More generally, we suggest that unbiased MEG based approaches can be used to identify non-obvious pathogenicity genes in complex multifactorial diseases.

## Results

### Integrative analysis of three datasets

**Fig 1:**
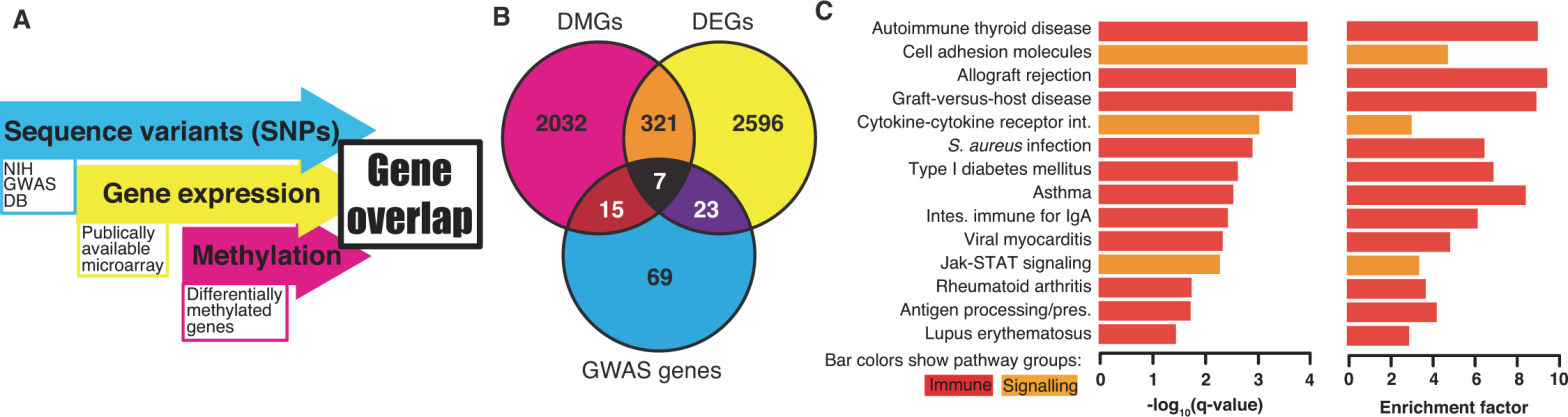
The RA multi-evidence gene set. (**A**) An overview of the omics data integration strategy; DB stands for database. (**B**) A Venn diagram shows the overlap between the differentially methylated genes (DMGs), differentially expressed genes (DEGs) and genome-wide associates study (GWAS) genes. (**C**) KEGG enrichment analysis of the RA multi-evidence gene set. The KEGG pathway enrichment analysis of GWAS, DMGs, and DEGs was performed using genes that were in 2 or 3 of the datasets. *P-*values were calculated using the hypergeometric distribution. The *P-*values we corrected for multiple testing to produce *q*-values. Pathways with *q*-values <0.05 are listed.

Genome-wide analysis often identifies many candidates for further investigation. However, selecting the most promising from hundreds, or even thousands, of candidates can be difficult. We have performed an integrative analysis of three genome-wide measures of rheumatoid arthritis: (i) genetic variation identified in genomic DNA, (ii) DNA methylation in RA FLS, and (iii) gene expression in RA FLS. Each dataset was individually analyzed to identify promising candidates (Fig 1A). Then we compared the results to identify candidates that were consistently found by two or three genome-wide measures to select the most promising candidates (see Material and Methods). As shown in Fig 1B, multiple genes overlapped between two of the three datasets (GWAS, DMGs, and DEGs). Seven genes were identified in the three-way intersect (*AIRE*, *CASP8*, *CSF2*, *ELMO1*, *ETS1*, *HLA-DQA1* and *LBH*), which was highly statically significant (*P*-value = 0.0001). Some are already known to be relevant to RA, such as *HLA-DQA1* [17] and *CSF2* [18]. The relationship between the triple evidence genes and RA for the others was less clear and was subjected to further functional analysis (see below).

### Pathway analysis of integrative analysis

Our previous DMG-focused study of KEGG pathways demonstrated significant enrichment in multiple pathways relevant to RA [4]. To assess the pathways implicated in RA using the integrative analysis of GWAS, DMGs, and DEGs, we performed a similar analysis for MEGs that were in 2 or 3 of the datasets. There are 366 MEGs with either 2 or 3 forms of evidence (S1 Dataset) that were used in KEGG pathway enrichment analysis (Fig 1C and S2 Dataset). As with the original DMG-alone analysis, the MEGs were also significantly 3.75-fold enriched in the KEGG ‘Rheumatoid arthritis‘ pathway (*q* = 1.70E-02, where *q* is a multiple-test corrected *P-value*) with 7 out of 89 genes: *ANGPT1*, *CSF2*, *CTLA4*, *HLA-DQA1*, *HLA-DQA2*, *HLA-DRA* and *HLA-DRB1*. These data support the notion that the MEGs are highly relevant to RA. Furthermore, at least four additional immunological pathways relevant to RA were also identified as significantly enriched in the MEGs. For example, the KEGG ‘Cell adhesion molecules (CAMs)' pathway is 4.80-fold enriched (*q* = 1.05E-04) with 13 out of 129 genes in the MEGs. The KEGG ‘Cytokine-cytokine receptor interaction’ pathway is 3.08-fold enriched (*q* = 8.74E-04) with 16 out of 248 genes labeled in the MEGs. The KEGG ‘Antigen processing and presentation’ pathway is 4.27-fold enriched (*q* = 1.78E-02) with 6 out of 67 genes labeled in the MEGs. The KEGG ‘Jak-STAT signaling pathway’ pathway is 3.43-fold enriched (*q* = 4.91E-03) with 10 out of 139 genes labeled in the MEGs. Thus, integrative analysis identified genes and pathways that could play a critical role in the pathogenesis of RA.

### Identification of a key gene in the triple evidence overlap: *ELMO1*

**Fig 2:**
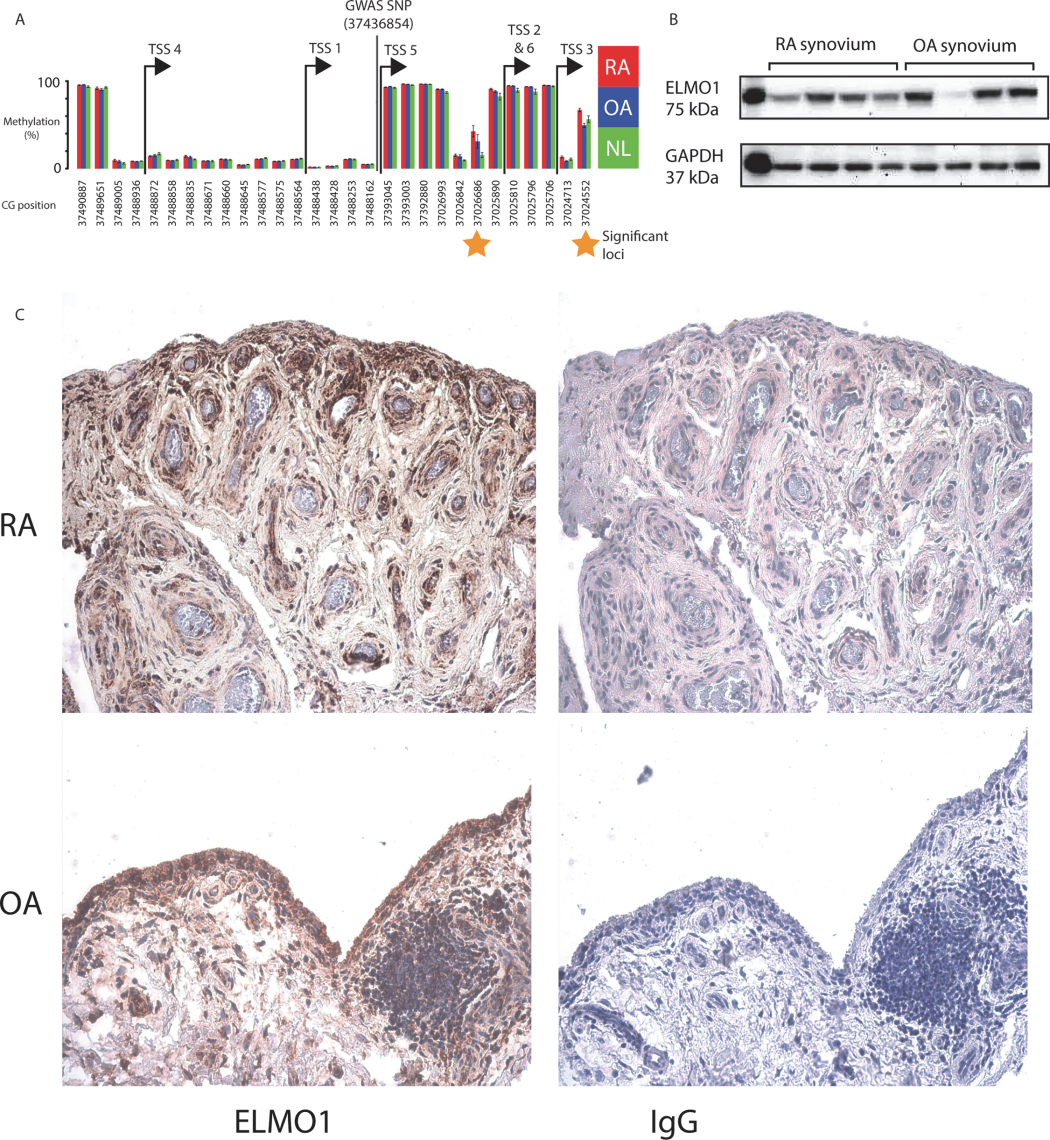
*ELMO1* methylation and expression in RA FLS and synovium. (**A**) A summary of DNA methylation at the *ELMO1* promoter regions from our previous study of FLS [4] and the location of the *ELMO1* RA GWAS SNP. The methylation levels of each of the CGs on the bead array that are within the *ELMO1* promoter region (-2500bps to +500bps from TSS) are shown. Error bars represent the standard error of the mean (SEM) and stars highlight significantly differentially methylated CGs. The locations of the TSSs are indicated with arrows and the transcript variant numbers of the RefSeq genes that are transcribed from that TSS are shown. Comparisons are shown between RA, osteoarthritis (OA) and normal (NL) FLS. (**B**) Western blots showing the expression of and GAPDH in RA and OA synovial tissue. First lane shows positive control, and subsequent lanes show *ELMO1* protein levels in whole RA and osteoarthritis (OA) synovium (n = 4 each). There was no significant difference in overall *ELMO1* expression. (**C**) Immunohistochemistry for *ELMO1* expression in RA and OA synovial tissue. Note prominent staining in synovial intimal lining and sublining perivascular regions (brown color). Osteoarthritis synovium had a similar distribution. Left panels shows anti-*ELMO1* antibody. Right panels shows control IgG. Tissues were lightly counterstained with hematoxlin. Original image at 200x magnification.

We identified seven MEGs that overlapped in the DMG, DEG, and GWAS datasets. Of these, we were particularly interested in the cytoplasmic engulfment protein, *ELMO1*, as being a promising candidate because of its putative role in cell migration [16]. Therefore, we examined its function more carefully. ELMO1 functions downstream of the phosphatidylserine receptor, BAI1, and forms a complex with DOCK1 and RAC1 [19–21]. Complex formation activates RAC1, a plasma membrane-associated small GTPase, leading to promotion of cell motility and engulfment. *ELMO1*-deficient knockout mice are viable but have decreased sperm production due to reduced phagocytic clearance of apoptotic germ cells, demonstrating the importance of *ELMO1* in Sertoli-cell-mediated engulfment [22]. The ELMO1, DOCK1, and RAC1 pathway has been implicated in: phagocytosis of Gram-negative bacteria by macrophages [23], T cell migration in primary lymphocytes [24], and actin cytoskeleton regulation during breast cancer metastasis [25]. Inhibition of RAC1 in RA FLS inhibits cell proliferation and invasions and demonstrates RAC1’s role in RA FLS aggressive behavior [26]. *ELMO1* is a complicated gene with seven gene models and four transcription start sites (TSSs) listed in the NCBI’s RefSeq database. Fig 2A shows that the significantly differentially methylated loci (DML) overlap the promoter regions of transcript variant 2, 3 and 6. The identified GWAS SNP (dbSNP ID = rs11984075; RA association *P-*value = 5.00E-08) is A to G variant and overlaps intron 1 of transcript variant 1 [27]. Based on these data, we subsequently evaluated whether *ELMO1* contributes to pathogenic behavior of RA FLS.

### *ELMO1* expression in RA synovium and FLS

Initial studies were performed to confirm that *ELMO1* is expressed by RA FLS. qPCR studies showed that it is constitutively expressed, as expected based on the transcriptome data. Also as predicted by increased promoter methylation in RA, *ELMO1* mRNA expression was lower in RA FLS than OA FLS (0.247±0.072.and 0.446±0.066, respectively; p<0.05). Of interest, short-term exposure to pro-inflammatory cytokines that are associated with RA (*IL-1* and *TNF*) did not affect *ELMO1* expression, which is not surprising since cytokine exposure requires at least 2 weeks to alter DNA methylation and chromatin remodeling [28,29]. Western blot also demonstrated ELMO1 protein expression in RA synovium. Expression was similar in RA and OA tissues, most likely because of the sublining cells account for much of the tissue ELMO1 protein and differential methylation has only been demonstrated in RA FLS (Fig 2B). Immunohistochemistry was then performed to localize ELMO1 protein in RA synovium. Fig 2C shows that immunoreactive ELMO1 is expressed in the synovial intimal lining, where FLS reside *in situ*. Sublining mononuclear cells also contained ELMO1.

### Role of *ELMO1* in FLS migration and invasion

**Fig 3:**
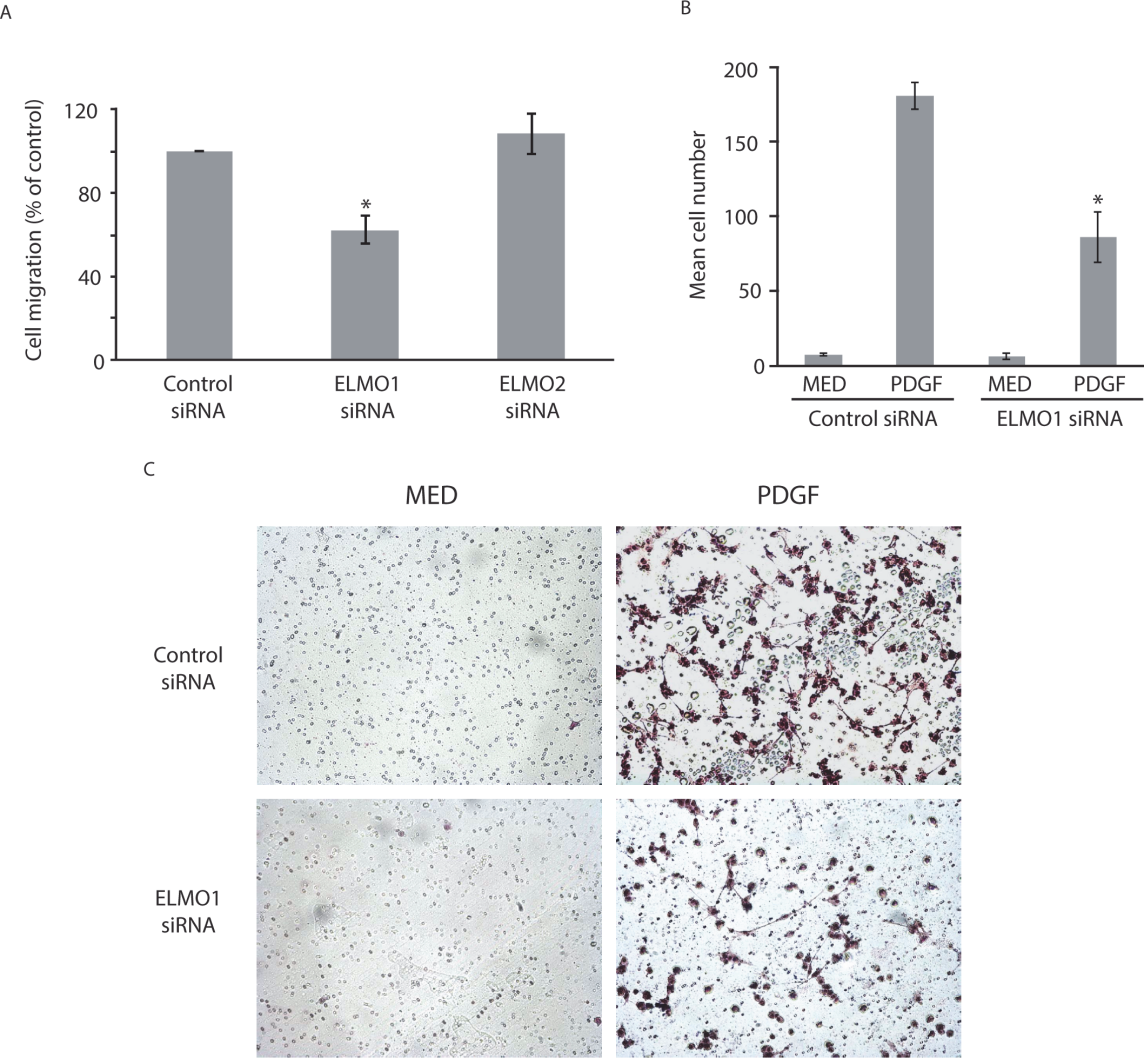
*ELMO1* promotes cell migration and invasion by RA FLS. (**A**) Cell migration in the wound closure assay shows that *ELMO1* knockdown with siRNA decreases cell migration whereas the control or ELMO2 siRNAs do not. Mean and SEM was calculated from 5 experiments for *ELMO1* and 3 experiments for ELMO2. (**B**) Cell invasion assay shows that *ELMO1* promotes the movement of FLS into extracellular matrix. Levels were calculated in the presence and absence (MED) of PDGF and with control and *ELMO1* siRNA. Mean cell number and SEM was calculated from 10x field-of view images and then normalized to control. (**C**) Fields of view showing FLS invading through a Martigel layer. Note the decreased number of invading cells when the FLS are pre-treated with *ELMO1* siRNA.

Because ELMO1 regulates RAC1 and, therefore, could modify cell movement, we determined if *ELMO1* deficiency suppresses cell migration. Fig 3A shows that *ELMO1* siRNA, which decreased *ELMO1* expression by >90%, significantly decreased cell migration in the wound closure assay. Control siRNA or siRNA specific for a related gene, namely *ELMO2*, had no effect. Cell invasion is another phenotypic feature of RA FLS and requires movement of cells into an extracellular matrix. Therefore, we determined if ELMO1 participates in FLS invasion into extracellular matrix. As shown in Fig 3B and 3C, *ELMO1* deficiency markedly decreased FLS invasion into Matrigel, a commonly used matrix that is comprised mainly of laminin.

### Regulation of RAC1 by ELMO1 in RA FLS

**Fig 4:**
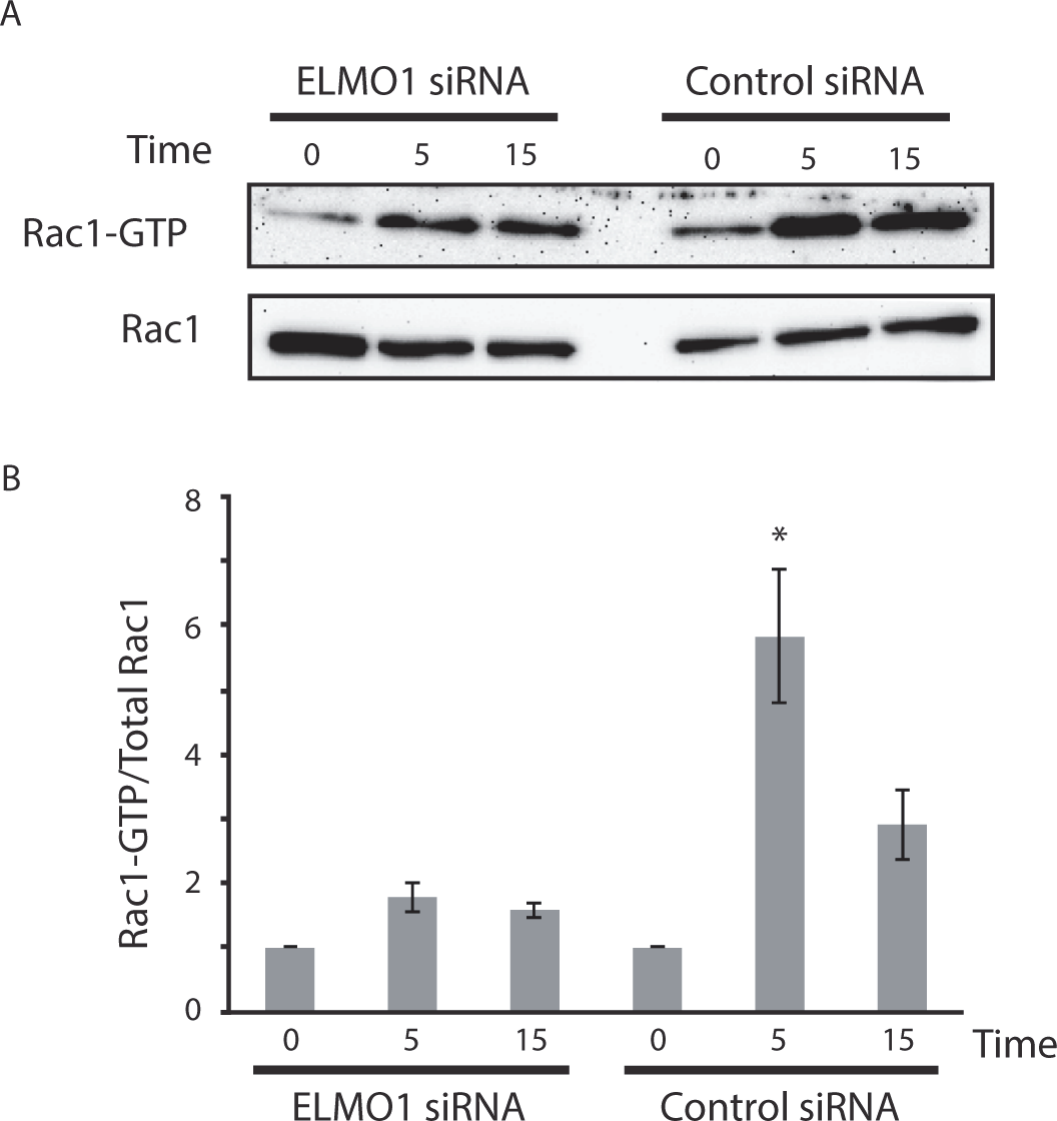
*ELMO1* regulates RAC1 in RA FLS. (**A**) Western blots showing RAC1 and Rac1-GTP levels in RA FLS following PDGF stimulation with *ELMO1* and control siRNA. RAC1 activation was determined as described in Material and Methods. Cells were assayed from 0 to 15 min after they were stimulated with PDGF. (**B**) Quantification of the RAC1 assay. Mean and SEM was calculated from 3 experiments. The ratio of Rac1 and Rac1-GTP in RA FLS following PDGF stimulation with *ELMO1* and control siRNA is shown and demonstrates decreased RAC1 activation when FLS are *ELMO1*-deficient.

Because ELMO1 likely regulates cell movement by interacting with DOCK1 and regulating RAC1 [30], we then evaluated whether ELMO1 deficiency suppresses RAC1 activation. Fig 4A and 4B, shows that ELMO1 deficiency markedly decreased PDGF-induced RAC1 activation, suggesting that this is how ELMO1 siRNA blocks migration and invasion.

## Discussion

One of the challenges in RA drug development is identifying robust and novel targets. To address this issue, we established an MEG strategy based on unbiased omics data. Because differential methylation can result in altered gene expression from expansion of repressive histone modifications [31], we did not make assumptions about the relationship between DNA methylation and gene expression. Thus, we identified numerous double-evidence genes that are dysregulated and might contribute to disease. Pathway analysis confirmed that these MEGs participate in immune responses considered relevant to RA and strongly support the theoretical underpinnings of this approach and provide evidence of *in silico* validation. As with our previous KEGG studies on DMGs [4], ‘Rheumatoid Arthritis‘, ‘Cell Adhesion‘, and other pathways indicate dysregulation or imprinting of RA FLS in a manner that could predispose to inflammatory disease and an aggressive RA phenotype.

Our MEG method of candidate gene prioritization is powerful as irrelevant genes that might be identified because of assay specific random changes, or biases, are largely removed from the final set of MEGs. Furthermore, the MEGs method produces a set of candidate genes that is manageable during further experimentally validation. Moreover, by combining genetic information from GWAS, which identifies variants that drive disease, with RA specific gene expression and DNA methylation information in FLS, we can assign disease- related genes to the tissue in which they drive pathogenesis. This is particularly relevant in RA FLS as there are no current RA therapies that target these cells.

When we narrowed our analysis triple-evidence genes, we identified seven genes that overlapped between all three datasets. A significant enrichment of genes in the triple-evidence category (*P*-value = 0.0001) was calculated using permutation analysis and suggests the genes are of high relevance to RA. Of these, *HLA-DQA1* and *CSF2* (which encodes *GM-CSF*) already had known roles in RA [17,18,32]. Furthermore, elsewhere we have validated another triple-evidence gene, *LBH*, as a regulator of cell cycle in RA FLS and another potential RA therapeutic target [33]. Herein, we present detailed experimental characterization of a gene previously unstudied in RA, namely *ELMO1*, on the basis of its potential role in regulating cell migration [21].

Initial studies showed that *ELMO1* is abundantly expressed in the inflamed synovium, including the synovial intimal lining *in situ*. Expression was lower in RA FLS, which potentially represents an inadequate compensatory mechanism to decrease synoviocyte activation through methylation. ELMO1 activates RAC1 signaling leading to the promotion of cell motility and engulfment [16,21,30]. To establish the role of *ELMO1* in RA FLS we first established that *ELMO1* is expressed in the RA synovium. While ELMO1 expression was higher in OA synovium, the synovium is a mixture of cell types and *in situ* difference in overall expression would not be expected. Next we showed *in vitro* that inhibiting *ELMO1* transcription with siRNA in RA FLS reduces cell migration and invasion. Knockdown required 90% effectiveness to show effect demonstrating that the modest difference in *ELMO1* expression between RA and OA is unlikely to be of any clinical effect. Finally, we confirmed that RAC1 GTPase activity is dependent on ELMO1, suggesting that in RA FLS ELMO1 promotes cell motility and invasion through RAC1 activation. Because FLS are largely responsible for cartilage damage in RA and can potentially migrate to other joints, blocking migration could have a major impact on disease progression [34,35]. However, these results do not rule out other potential roles of *ELMO1* in RA. For example *EMLO1* might promote migration of inflammatory immune cells to inflamed joints [24].

These data support the MEG-derived hypothesis that *ELMO1* contributes to the pathogenesis of RA and supports the MEG methodology of prioritizing disease-related genes. Previously studies have shown that ELMO1:DOCK2 complex formation is essential for RAC signaling to promote cell migration [36]. Furthermore, a crystal structure of the ELMO1:DOCK2 complex suggests that an inhibitor could disrupt complex formation by binding to the interaction face of DOCK2 [36]. Thus, the function of ELMO1 creates the potential to treat RA through inhibition of ELMO1:DOCK2 complex formation or perhaps focusing on other proteins in the same pathway.

Taken together, we have shown that the MEG approach can successfully go from *in silico* integration of multiple genome-wide assays to *in vitro* validation of a potential RA pathogenicity gene. The ability to target either the MEG or another gene in the pathway provides novel ways to identify and prioritize drug discover efforts. Further work in this direction will expand upon this by characterizing the role of the other RA MEGs and by using newer dataset to expand the set of MEGs. The expansion of our datasets could be carried out by replacing existing datasets with higher quality data, e.g. the microarrays using to measure gene expression could be augmented by RNA-seq. Furthermore, the results of additional assays could be incorporated e.g. ChIP-seq could be used to identify genes with different chromatin modification patterns or differentially active regulatory enhancer regions [37]. These expansions could be complemented by new epigenomic analytical methods that allow regulators of epigenomic changes to identified [38]. Beyond RA, we suggest unbiased MEG approaches should be used when studying complex multifactorial diseases, such as cancers and other immune diseases.

## Materials and Methods

### Preparation of human synovial tissue and FLS

This study was approved by the Institutional Review Board of University of California, San Diego School of Medicine (Scripps and UCSD Human Research Protection Programs) and written informed consent was obtained from all participants. Synovial tissue was obtained from patients with OA and RA at the time of total joint replacement or synovectomy as previously described [39]. The diagnosis of RA conformed to American College of Rheumatology 1987 revised criteria [40]. The samples were either processed for cell culture or snap froze for immunohistochemistry. For preparation of FLS the synovium was minced and incubated with 1 mg/ml collagenase type VIII (Sigma Chemicals, St. Louis, MO) in serum-free RPMI 1640 (Gibco BRL, Grand Island, NY) for 1h at 37°C, filtered, extensively washed, and cultured in DMEM (Gibco BRL) supplemented with 10% FBS (Gemini Bio Products, Calabasas, CA), penicillin, streptomycin, gentamicin, and glutamine in a humidified 5% CO_2_ atmosphere. Cells were allowed to adhere overnight, non-adherent cells were removed, and adherent FLS were split at 1:3 when 70–80% confluent. FLS were used from passage 3 through 9 during which time they are a homogeneous population of cells (<1% CD11b positive, <1% phagocytic, and <1% FcγRII and FcγRIII receptor positive). FLS were cultured and used at 80% confluence. Cells were synchronized in 0.1% FBS for 24h before the addition of the appropriate stimulus.

### Processing omics data

We collected three types of RA genome-wide datasets: (i) genetic variation data from GWAS, (ii) epigenetic data from DNA methylation bead arrays, and (iii) gene expression data from microarrays. To establish a set of genes that have been implicated in RA from GWAS we downloaded a catalog of published GWAS [41,42] Then we extracted all studies relating to RA susceptibility. Furthermore, we added RA GWAS genes from a recent meta-analysis of over 100,000 cases and controls [43]. In total 114 GWAS genes were identified.

The set of DMGs were taken from our previous study [4] (GEO ID = GSE46364). Briefly, methylation beads arrays were used to measure the methylation levels of 485,000 methylation sites in FLS of patients with RA, OA and normal (NL). Each sample type had the following number of bead arrays: 11 RA, 11 OA and 6 NL. To identify loci that were differentially methylated between samples Welch’s *t*-test was used to calculate *P-*values. Loci was considered differentially methylated if they have a multiple testing corrected *P*-value (referred to as *q-*values) <0.05 and sample mean difference >0.1. Then DMGs were identified as those that have differentially methylated loci within their promoter region (-2500bps to +500bps from a genes TSS). In the original study the RA samples were compared to the other samples in several different ways. We opted to use the most robust analysis, which was the combined set of DMGs as it includes all identified DMGs (2,375).

To establish a set of differentially expressed RA genes we downloaded a set of microarrays from the FLS of patients with RA, OA and NL [7] (GEO ID = GSE29746). Each sample type had the following number of microarrays: 9 RA, 11 OA and 11 NL. The microarray data were initially processed using Agilent Technologies Feature Extraction Software. Then the significant of differential expression between samples was calculated by using Welch’s *t*-test. We chose to use Welch’s *t*-test as the samples may have unequal variance. To establish a set of DEG we took all genes with greater than 2-fold change in expression and *P*-value <0.05. We did not use a *P*-value that had been corrected for multiple testing as an uncorrected *P*-values as used in the datasets original publication, as the intra-sample variation was too great. If multiple probes for the same gene existed they were treated separately rather than being averaged. Thus, if one probe is differentially expressed, the gene is considered as differentially expressed. We took this approach as the different probes may relate to different exons and averaging may mask differential expression brought about alternate splicing/transcription start sites. As the Agilent arrays use 60-mer probes they have high specificity making such an approach feasible. The RA, OA and NL samples were compared in same way the methylation samples were compared. This resulted in a combined set of 2,947 DEGs.

### Calculating the significance of the gene set intersect

We wished to calculate the significance of the level of overlap between the three RA omics datasets. Thus, we devised a permutation test that randomly reselected sets of identifiable genes from each of the datasets. To do this, we started by making lists of all the genes that could have been discovered in each of the three analyzes. For the DMGs, the entire set of identifiable genes is those genes that loci within their promoters that are measured on the bead array. For the DEGs, it is all genes that have probes on the microarray. For GWAS genes, the situation is more complicated as GWAS database is constructed from many studies that use a mixture of platforms. Thus, we took all the genes that are reported in the GWAS database, regardless of the condition that they are linked to. During each round of the permutation analysis genes were randomly selected from each of the potentially identifiable sets. The number of selected genes was equal to the number of genes identified in that datasets analysis e.g. for DMGs 2,375 genes were randomly selected. Then the overlap between the three dataset was calculated. This procedure was repeated 10,000 times. Then a distribution was created and *P*-value calculated.

### Pathway analysis

KEGG pathway enrichment analysis of the double evidence gene sets was performed using the same methodology as our previous study [4].

### Quantitative real-time PCR

Cells were grown until 70–80% confluence and subsequently serum starved (0.1% FBS/DMEM) for 24h for synchronization as previously described [44]. RNA isolation and RT-PCR were performed as previously described [45]. The threshold cycle (Ct) was determined for each sample using GeneAmp software. The ratio between the gene of interest and GAPDH cell equivalents (relative expression units, REU) is reported.

### Western blot analysis

Synovial tissue protein was extracted using PhosphoSafe buffer (Novagene, Madison, WI) supplemented with Complete Proteinase Inhibitors (Roche Applied Science, Indianapolis, IN, USA). RIPA buffer was used for protein extraction from synovial tissue. The protein concentrations of tissue and FLS were determined using the Micro BCA protein assay kit (Thermo Scientific, Rockford, IL). Samples containing 25 μg of protein from cultured FLS or 50 μg of protein from synovial tissue were resolved on Invitrogen (Carlsbad, CA, USA) NuPage 4 to 12% precast gels and transferred to a PVDF membrane. Membranes were developed with Immun-Star Western ECL substrate (Bio-Rad, Hercules, CA, USA) and imaged on VersaDoc imaging system (Bio-Rad), using QuantityOne software (Hercules, CA, USA) for image capture and densitometry.

### Cell migration assay

For the wound closure motility assay, FLS were plated in 6 well plates at 70–80% confluence and serum starved (0.1% FBS/DMEM) overnight [46]. A linear wound was created using a 1 ml micropipette tip then washed three times with starving media to remove unattached cells. Cells were incubated with PI3K inhibitors or DMSO for 1 h then 0.1% FBS containing media +/- PDGF-BB (10 ng/ml) was added_._ Light microscopy images were taken immediately 0 and at 36 hours after wounding. At the end of the experiment cells were fixed and stained using Hemacolor staining kit (EMD Millipore, Billerica, MA). Light microscope images for three locations of marked wound were taken and migrated cells were counted using NIH ImageJ software. The number of migrated cells was normalized to media control and this value represents the migration index.

### Invasion assay

The BD BioCoat Growth Factor Reduced Matrigel Invasion Chambers (8 μm pore diameter; BD Biosciences) were used to evaluate invasion through a Matrigel layer as previously described [46]. To measure cell invasion, 2.5 x 10^4^ cells in medium containing 0.1% BSA were added to the transwells. Medium supplemented with PDGF (25 ng/ml) was used as an attractant in the lower chamber. After 24 h the cells that invaded through the matrix were fixed and stained with Hemacolor staining kit (EMD Millipore, Billerica, MA). The number of invading cells was averaged from three 10x field-of view images and normalized to control.

### RAC1 GTPase activity assay

Activated RAC1 was detected were using RAC1 assay reagent (GST-PAK1-PBD on glutathione-Sepharose Beads) according to the manufacturer’s instructions (Cytoskeleton, Denver CO). GST-PAK1-PBD specifically binds to the GTP-bound form of Rac1. RA FLS were plated in 10 cm dishes at 50–70% confluence, serum starved with DMEM for 48 h. Cell were pretreated with PI3K inhibitors or DMSO for 1 h followed by PDGF (10 ng/ml) stimulation. Samples were processed according to the manufacturer’s instructions. RAC1-GTP and total RAC1 protein levels were visualized by Western Blotting.

### Immunohistochemistry of synovium

Staining protocols were performed as previously described [47]. Five micron cryosections of synovial tissue were cut, fixed in cold acetone for 10 min and incubated with the appropriate Abs overnight at 4°C. Isotype matched antibodies served as a negative control. Endogenous peroxidase was depleted with 0.3% hydrogen peroxide and sections then stained with secondary antibodies (Vector Laboratories, Burlingame, CA). The signal was developed using diaminobenzidine and sections were counterstained with hematoxylin.

### siRNA transfection of FLS

5X10^5^ FLS (passage 4–6) were transfected with 1–3 μg targeting *ELMO1*, *ELMO2* or scramble (sc) control Smartpool siRNA (Dharmacon, Lafayette, CO), using normal human dermal fibroblast Nucleofector kit, according to the manufacturer’s instruction (Amaxa, Gaithersburg, MD).

### Statistical analysis for FLS biology

Statistics were performed using the paired Student’s *t*-test. A comparison was considered significant if *P*<0.05. In the cell migration assay an unpaired Student’s *t*-test was.

## Supporting Information

S1 Dataset An excel file containing the complete list of multi-evidence genes. (XLSX)

S2 Dataset An excel file containing the complete KEGG enrichment analysis. (XLSX)
