# Correction: Integrative Omics Analysis of Rheumatoid Arthritis Identifies Non-Obvious Therapeutic Targets

**DOI:** 10.1371/journal.pone.0136334

**Published:** 2015-08-19

**Authors:** John W. Whitaker, David L. Boyle, Beatrix Bartok, Scott T. Ball, Steffen Gay, Wei Wang, Gary S. Firestein

There are errors in the Funding section. The correct funding information is as follows: This project was supported by a grant from the Rheumatology Research Foundation.
